# The Present Position of Nursing

**Published:** 1893-07-08

**Authors:** 


### The Hospital

An Institutional Journal of

**Science, Medicine, Nursing, and Philanthropy.**

*For Hospitals, Asylums, Infirmaries, Dispensaries, Medical Practitioners, Students, Nurses, and the Charitable Public.*

Vol. XIV.—No. 354.] July 8, 1893.

### The Present Position of Nursing.

The holding of the first International Nursing Congress at Chicago, the decision of the Privy Council in relation to the application of the Royal British Nurses' Association for a charter, and the manifesto in regard to that decision which has been issued by Miss Florence Nightingale and the managers, teachers, and matrons of the chief London hospitals, mark an epoch in the history of nursing. The controversy which arose, owing to the impatience of a few ardent spirits, generated a heat which all sensible people regret. Let us hope that the three great events in the history of nursing which we have set forth may lead to the cordial union of all who are interested in the welfare and progress of trained nurses. One useful purpose has been served by the controversy which we hope is now at an end. It has opened the eyes of all who are connected with the training of nurses in the United States to the dangers of premature action, and it has promoted unity of purpose and hearty co-operation amongst all sections of American nursing opinion. It is our hope that similar wisdom may be manifested by those who are responsible for the training and management of nurses throughout Great Britain, as well as by the nurses themselves.

The International Nursing Congress will clear away much misconception regarding the position and progress of the American nurse-training schools. It has been held by many people in this country that the administration of United States hospitals, so far as the organisation of the nursing and the training of nurses are concerned, is much behind the best of our hospitals. That the control is vested too much in the medical superintendents, who keep the matrons and superintendents of nurses in a very subordinate position, which leads to an absence of thoroughness in the training given to the nurses. These views are not in accordance with the facts. A careful investigation which we have recently made of the system of training nurses, and the position held by the matrons and lady superintendents in American hospitals, proves beyond dispute that in many respects the training is far more thorough and extended in the best hospitals of the United States, than it is in British hospitals. It is true that at several of the American schools nurses may graduate at the expiration of a two years' course, but that course represents the completest training, and is characterised by a thoroughness which it would be difficult to excel. The lady superintendents and matrons of the great hospitals possess, on the whole, more rather than less authority than is delegated to the same officials in this country. It has further been contended that American nurses are mainly recruited from the more educated classes, who go as probationers for the sake only of qualifying themselves to work independently as private nurses to the detriment of the permanent hospital staff. We have very thoroughly tested this contention, and find that, on the whole, the type of nurses in America differs very little, if at all, from that which prevails in British hospitals. For all these reasons it is but just that at least equal weight should be given to the views of American authorities, teachers, and nurses, as to those held by our own people.

We have said that the controversy in this country, which so many have deplored, has had a marked effect for good in America. It has taught our cousins to think well before encouraging new departures which may in the long run tend rather to retard than to advance the progress of skilled nursing. The question of registration in all its bearings was carefully considered by the representatives who assembled in such numbers at Chicago. It was the unanimous feeling that at the present time it would be unwise to attempt to establish a general register, and that before any such steps could be taken it was essential that each nurse-training school and all the nurses which had graduated from it should be united into an alumni association, or society, that the needs, views, and wishes of the whole body might be accurately ascertained, and so the nurses might have an opportunity of learning how to organise themselves in the best interest of their profession. It was further held to be desirable to establish an association of matrons and lady superintendents, who will thus be enabled to compare notes, and ultimately to ascertain how far it may be possible to secure something like uniformity in the curriculum and teaching of the American nurse-training schools. When the alumni and superintendents' associations have been completely organised, and so soon as nurses and superintendents have learned to conduct their business in an orderly and businesslike way, each alumni association will be asked to send two representatives to a conference of all the schools, who will be armed with definite instructions as to the views of their school upon the many questions which affect the well-being and progress of the nursing world. Such a conference, and such a conference alone, will be competent to determine what steps it is desirable to take in the best interests of trained nurses in order to provide reasonable security and efficiency for all. We cannot but congratulate our American cousins upon the wisdom which they have displayed in this matter, and we believe their action to be well worthy of the careful consideration of every nurse, as well as of the teachers and authorities of the nurse-training schools in the United Kingdom.

Let us briefly consider the position of trained nurses in Great Britain to day. They have absolute protection against pecuniary risks and distress through the ordinary and benevolent departments of the Royal National Pension Fund. Private nurses can secure their earnings for their own use by joining the Nurses' Co-operation. The Privy Council has granted a Charter to the Royal British Nurses' Association, but has declined to give it authority to establish a chartered register. Any nurse who chooses to pay the fees and subscriptions which the Association demands may become a member, and may enter her name upon "the list of persons who may have applied to the Royal British Nurses' Association to have their names entered thereon as nurses, and whom the Association may think fit to enter thereon from time to time." Our readers are aware that the list in question already contains the names of a large number of uncertificated nurses, and that any trained nurse who is foolish enough to pay a fee, and to enter her name thereon, must be prepared to accept any disadvantages which may accrue to her from this circumstance. She must also bear in mind, as Miss Nightingale points out, that no professional privileges will be obtained by the nurses whose names appear upon this list. The list will have nothing in common with the legal registers of the medical or other professions, but will simply be a list of nurses published by the Royal British Nurses' Association. Further, no nurse whose name appears on the list will have any right to use the title of *registered nurse*. In other words, the practical advantages which a nurse will gain by the pecuniary payments she must make in order to get upon the list of the British Nurses' Association are not apparent. Such payments will probably prove a somewhat expensive investment, seeing that they procure her no adequate professional privileges, no protection or discipline as in the case of the medical register, nor any right to a recognised title conferring honour and status upon its holder. In other words, the granting of a charter to the Royal British Nurses' Association has not created a legally authorized register of trained nurses, and so whilst the Association is at liberty to promote the objects set forth in its charter for its own benefit, neither the public nor trained nurses must expect to gain practically much, if anything, from the authority relegated to the British Nurses Association by its charter. We purposely refrain from emphasizing the legitimate objection which all good nurses must have to the charter as it now stands, as Miss Catherine J. Wood has rightly emphasized this point in a letter which will be found in another portion of our present issue.

Finally, what course should the nurse training schools, the matrons and lady superintendents, and the trained nurses of Great Britain now pursue? Their position to-day is one of entire independence, and they may maintain and strengthen that independence by keeping their names off "the list" of the R. B. N. A., and adopting a similar attitude to that taken up by our American cousins. It is undoubtedly desirable that all the nurses who have been granted certificates by a nurse-training school should be associated together in an independent alumni association. It is also desirable that these alumni associations should be instituted and managed by the nurses themselves, with such counsel as they may seek at the hands of their matrons, lady superintendents, and school authorities. We are further of opinion that the time has arrived when it is desirable that the matrons and lady superintendents should form themselves into a separate association, that they may by conference and co-operation secure uniformity and efficiency of training throughout the country. We have already advocated the formation of an association of nurse-training schools, not necessarily for the purpose of immediate and active work, but to enable the authorities to whom the country is indebted for the present army of trained nurses to confer whenever necessary upon any point of importance, and to gradually learn to act together whenever such a course will promote the advancement of skilled nursing and adequate instruction. In making these suggestions we are anxious to avoid controversy, and we venture to hope that in future everybody will recognise the duty of respecting the honesty of the motives of those who differ from them, as well as the wisdom of promoting councils of peace in connection with so noble a calling as that of a nurse.

